# Diagnostic Accuracy of MRI for Orbital and Intracranial Invasion of Sinonasal Malignancies: A Systematic Review and Meta-Analysis

**DOI:** 10.3390/jcm13247556

**Published:** 2024-12-12

**Authors:** Umida Abdullaeva, Bernd Pape, Jussi Hirvonen

**Affiliations:** 1 Department of Radiology, Tashkent City Branch of the Republican Specialized Scientific and Practical Medical Center of Oncology and Radiology, Tashkent 100054, Uzbekistan; 2 Department of Biostatistics, University of Turku and Turku University Hospital, 20521 Turku, Finland; 3 School of Technology and Innovations, University of Vaasa, 65101 Vaasa, Finland; 4 Department of Radiology, Faculty of Medicine and Health Technology, Tampere University Hospital and Tampere University, 33520 Tampere, Finland; jussi.hirvonen@utu.fi

**Keywords:** paranasal sinus neoplasm, magnetic resonance imaging, neoplasm invasion, systematic review, meta-analysis

## Abstract

**Background/Objectives**: In this study, we review the diagnostic accuracy of magnetic resonance imaging (MRI) in detecting orbital and intracranial invasion of sinonasal malignancies (SNMs) using histopathological and surgical evidence as the reference standard. **Methods**: A systematic search of studies in English was conducted in MEDLINE and Embase, limited to articles published since 1990. We included studies using preoperative MRI to detect the intracranial and orbital invasion of SNMs, with histological or surgical confirmation as the reference standard, and reported patient numbers in each class as required to assess diagnostic accuracy. The outcome measures were sensitivity, specificity, positive predictive value (PPV), and negative predictive value (NPV). Heterogeneity was assessed with the Higgins inconsistency test (*I*^2^). **Results**: Seven original articles with 546 subjects were included in the review, with six included in the meta-analysis. The pooled overall accuracy for orbital invasion was higher at 0.88 (95% CI, 0.75–0.94) than that for intracranial invasion at 0.80 (95% CI, 0.76–0.83). The meta-analytic estimates and their 95% confidence intervals were as follows for intracranial/orbital invasion: sensitivity 0.77 (0.69–0.83)/0.71 (0.40–0.90); specificity 0.79 (0.74–0.83)/0.91 (0.78–0.97); PPV 0.76 (0.64–0.85)/0.78 (0.61–0.88); and NPV 0.82 (0.72–0.89)/0.90 (0.63–0.98). Substantial heterogeneity was observed in the Higgins inconsistency test (*I^2^*) for orbital invasion (84%, 83%, and 93% for sensitivity, specificity, and NPV, respectively). **Conclusions**: MRI yielded moderate-to-high diagnostic accuracy for intracranial and orbital invasion, despite some limitations leading to false diagnoses. Loss of the hypointense zone on postcontrast MRI was found to predict dural invasion. Infiltration of the extraconal fat beyond the periorbita was found to be an MRI feature of orbital invasion.

## 1. Introduction

Sinonasal malignancies (SNMs) are a rare but diverse group of tumors with a reported prevalence of 3% among all head and neck tumors [1,2]. Among tumors extending to the skull base and orbit, sinonasal carcinomas rank first, with the most common histological types being squamous cell carcinoma (SCC) and sinonasal undifferentiated carcinoma [3,4]. The incidence of orbital involvement in SNMs varies between 30 and 82%, depending on the tumor site and use of different criteria to define “orbital invasion” [5]. The presence of dural and orbital invasion of SNMs upgrades the tumor stage, worsens prognosis, and significantly changes treatment management [1,6,7,8,9].

Careful pretreatment assessment of the orbital and intracranial involvement of SNMs is necessary to develop an optimal treatment strategy [6,7,10]. Severe orbital and dural invasion are contraindications for endoscopic endonasal tumor resection [11]. Computed tomography (CT) and MRI are the principal radiologic modalities used to assess tumor invasion [7,12]. CT is able to accurately visualize the bony structures at the skull base and assess orbital wall defects but is limited in distinguishing pressure erosion from underlying tumor invasion of the periorbita/orbital fat or cribriform plate [12,13,14].

Due to its excellent soft tissue discrimination, MRI is generally considered a superior imaging tool for evaluating orbital and skull base involvement and preoperative tumor staging [15,16]. The diagnostic accuracy of MRI in assessing the orbital and intracranial invasion (ICI) of tumors varies widely among different studies. Studies provide different MRI features and criteria for evaluating tumor invasiveness and use various reference methods [6,7,12,13,15,17,18].

Difficulties in assessing invasion still exist in terms of recognizing periorbital continuity and distinguishing between reactive dural enhancement and infiltration [1,3]. A limited number of studies have investigated orbital invasion, with no consensus regarding the radiological criteria for determining orbital invasion and orbital preservation. Therefore, such studies report false-positive (FP) or false-negative (FN) results [7,12,13,15,16].

In previous diagnostic accuracy studies examining dural and orbital invasion, the majority of tumors were of the malignant sinonasal variety. Despite previous diagnostic accuracy studies on the subject, no studies have been conducted to determine the pooled diagnostic performance of MRI for orbital and intracranial invasion of SNMs to identify the predictors of invasion.

Therefore, we conducted this study to review the evidence for the diagnostic accuracy of MRI in detecting the orbital and intracranial invasion of SNMs using histopathological and surgical evidence as the reference standard.

## 2. Materials and Methods

This systematic review protocol was pre-registered in the Prospective Register of Systematic Reviews (PROSPERO) under registration number CRD42024492090. This work was performed following the updated Preferred Reporting Items for Systematic Reviews and Meta-Analyses (PRISMA) guidelines [19].

### 2.1. Literature Search

**Figure 1:**
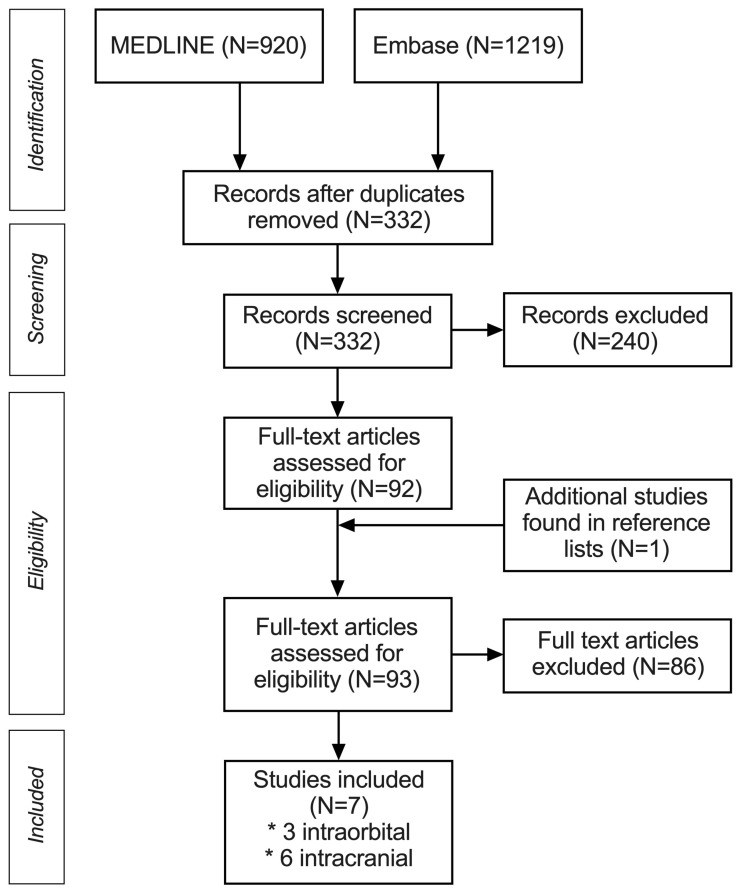
PRISMA flowchart of the systematic review and meta-analysis. * Four studies assessing the intracranial invasion, one study evaluating orbital invasion and two studies evaluating both types of invasions of sinonasal malignancies were found.

We comprehensively searched for the orbital and intracranial invasion of sinonasal malignant tumors in the MEDLINE and Embase databases according to the PIRO (population, target condition, index test, reference test, and outcome) strategy. We used both MeSH terms and text words during the literature search (Table S1). The reference lists of relevant studies were also searched (Figure 1). Near the end of the study, we conducted an additional literature search. However, no recent articles were found on this subject (20 August 2024).

### 2.2. Inclusion and Exclusion Criteria

Previous studies were searched using the MEDLINE and Embase databases. Relevant studies were identified based on the following inclusion criteria: (1) published in English from 1 January 1990 to 31 December 2023; (2) included patients of any age or gender with sinonasal malignant tumors; (3) the presence of MRI (with and without contrast enhancement) suggesting orbital and/or intracranial invasion of the sinonasal malignant tumors; (4) presented histological or surgical confirmation of tumor invasion; (5) patient numbers in each class reported with sufficient detail to calculate diagnostic accuracy (sensitivity, specificity, PPV, NPV, and accuracy). We excluded case report studies and studies that evaluated benign sinonasal tumors.

### 2.3. Data Extraction Strategy

Two radiologists (U.A., J.H.) independently screened the titles and abstracts. The same authors performed full-text screening and extracted data from the studies that met the eligibility criteria. The authors discussed any disagreements during the screening and data extraction steps to reach a consensus. The data extracted from relevant studies are presented in tables.

### 2.4. Study Quality Assessment

The QUADAS-2 [20] tool was used to assess the quality of the included studies independently via U.A. and J.H. across four domains (Tables S2 and S3). The final decision on the quality of the studies was made via consensus between the authors.

### 2.5. Data Synthesis and Presentation

The relevant studies were qualitatively assessed, considering the following factors: (1) diagnostic accuracy of the MRI on evaluation of the intracranial and orbital invasion of the SNMs; (2) primary tumor histology and location; (3) MRI techniques and protocols; (4) MRI features of intracranial invasion by sinonasal malignancies; and (5) MRI features of orbital invasion by sinonasal malignancies.

### 2.6. Statistical Analysis

True positives (TP), false positives (FP), true negatives (TN), and false negatives (FN) were evaluated in every study. We calculated the estimates of sensitivity (TP/[TP + FN]), specificity (TN/[TN + FP]), PPV (TP/[TP + FP]), and negative predictive value (NPV) (TN/[TN + FP]). Meta-analytic estimates were obtained by applying the random effects model by DerSimonian and Laird [21] to logit transformed accuracy, sensitivity, specificity, and predictive values. The analyses were performed on SAS System, version 9.4 for Windows (SAS Institute Inc., Cary, NC, USA), making use of the algorithm described in [22].

Heterogeneity was assessed using the *I*^2^ statistic by Higgins and Thompson [23], which estimates the proportion of the variance in study estimates that is due to heterogeneity rather than sampling error. In meta-analyses like ours, with a small number of studies, *I*^2^ is known to be imprecise, with possible bias [24]. When we found substantial heterogeneity (*I*^2^ > 80%), it could always be traced back to a single study estimate deviating from the others. In such cases, we performed sensitivity analyses by removing the study in question.

## 3. Results

### 3.1. Study Selection

After a thorough full-text review, seven studies met the inclusion criteria and were included in this systematic review; these studies analyzed a total of 546 patients with SNMs (Figure 1). Four studies [6,14,25,26] assessed the intracranial invasion of SNMs; one assessed orbital invasion [12,16]; and two studies [7,15] evaluated both types. Due to the lack of FPs and FNs in one study [14], only six studies were included in the meta-analysis.

In the meta-analysis, we included five studies [6,7,14,15,25,26] incorporating 420 patients with sinonasal tumors in which ICI was assessed via MRI and surgery/histopathology (mean age: 56.8 years). In total, four studies [7,15,25,26] were retrospective, and one [6] was prospective (Table 1). Meerwein et al. [15] provided data from two radiologists’ reports; these were included separately in the meta-analysis for completeness.

We found only three retrospective studies [7,15,16] suitable for analyzing orbital invasion in 356 patients, for whom tumor invasion was assessed via MRI and surgery/histopathology (mean age: 61.7 years) (Table 1). Studies not included in the systematic review are presented in Table S4.

### 3.2. Diagnostic Performance

The sensitivity of the studies with ICI ranged from 0.62 to 1.00 and specificity from 0.33 to 1.00, with median values of 0.79 and 0.82, respectively. PPV ranged from 0.61 to 1.00 and NPV from 0.50 to 1.00, with medians of 0.87 and 0.79, respectively. Due to the lack of FP and FN results, some studies reported a PPV [14,25] and NPV [14] of 1.00.

The sensitivity of the studies with orbital invasion ranged from 0.29 to 0.91 and specificity from 0.71 to 0.97, with median values of 0.77 and 0.93, respectively. PPV ranged from 0.50 to 0.86 and NPV from 0.47 to 0.96, with medians of 0.78 and 0.95, respectively.

**Figure 2:**
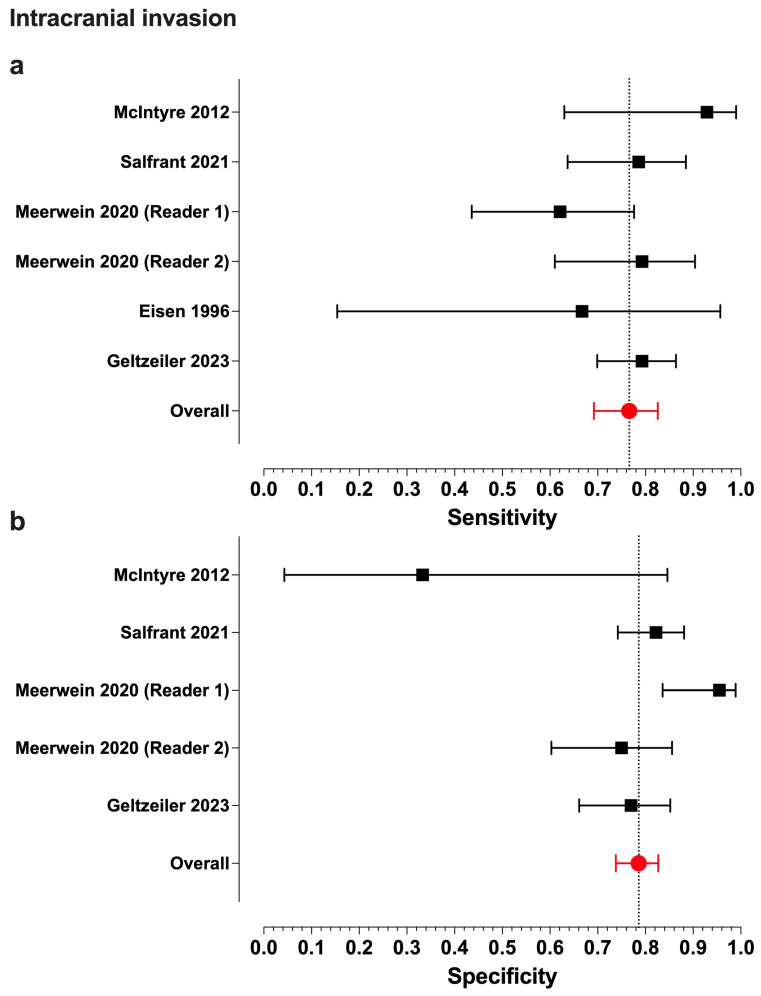
Forest plots of the sensitivity (**a**) and specificity (**b**) values from individual studies assessing intracranial invasion and their model-based estimates. The bars represent 95% confidence intervals, and the dotted lines represent the model-based estimates. Data shown from original publications: (**a**) [6,7,15,25,26], (**b**) [6,7,15,26].

**Figure 3:**
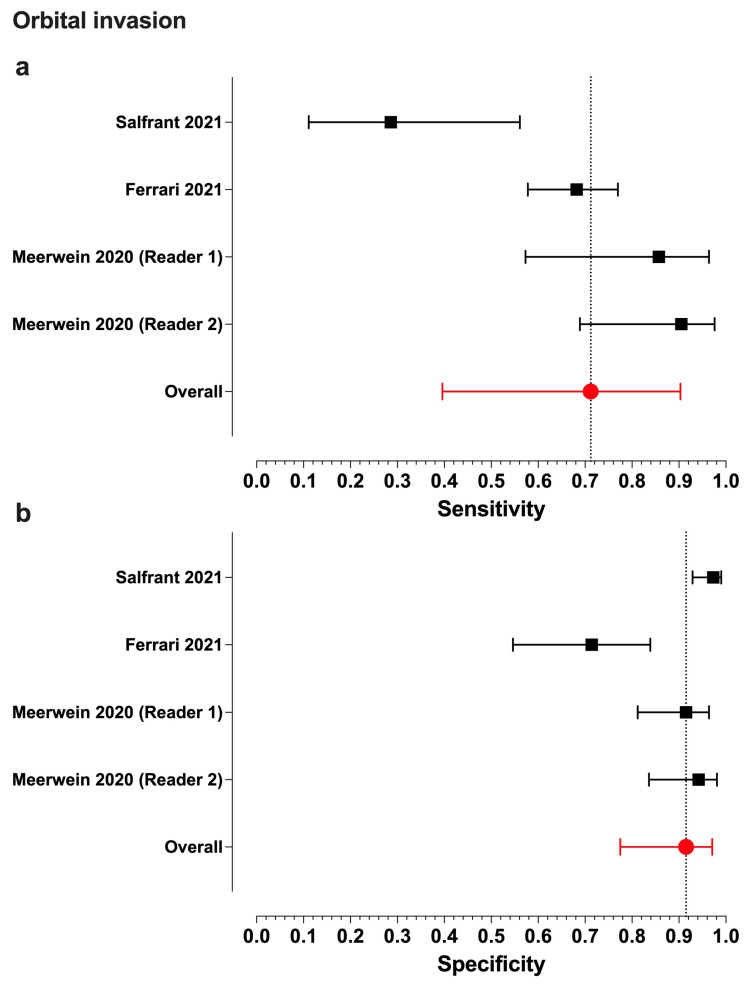
Forest plots of the sensitivity (**a**) and specificity (**b**) values from individual studies assessing orbital invasion and their model-based estimates. The bars represent 95% confidence intervals, and the dotted lines represent the model-based estimates. Data shown from original publications: (**a**,**b**) [7,15,16].

Meta-analytic estimates and their 95% confidence intervals were as follows for intracranial/orbital invasion: overall accuracy, 0.80 (0.76–0.83)/0.88 (0.75–0.94); sensitivity, 0.77 (0.69–0.83)/0.71 (0.40–0.90); specificity, 0.79 (0.74–0.83)/0.91 (0.78–0.97); PPV, 0.76 (0.64–0.85)/0.78 (0.61–0.88); and NPV, 0.82 (0.72–0.89)/0.90 (0.63–0.98) (Figure 2 and Figure 3).

We found statistically moderate heterogeneity in ICI for PPV (*I*^2^ = 64.3%, (5.3–86.3%)), alongside substantial heterogeneity in orbital invasion for sensitivity (*I^2^* = 83.9%, (41.8–92.0%)), specificity (*I^2^* = 83.0%, (63.0–94.0%)), and NPV (*I^2^* = 93.1%, (89.8–97.4%)).

Including only a small number of studies in a meta-analysis inevitably results in uncertainty regarding the true heterogeneity measure *I^2^* [24]. In our case, this is particularly evident in its confidence interval for PPV in ICI. While its lower bound of 5.3% falls within the low heterogeneity range, its upper bound of 86.3% suggests substantial heterogeneity.

Heterogeneity in orbital invasion was solely caused by Salfrant et al. for sensitivity and Ferrari et al. for specificity and NPV. Disregarding the relevant study in the respective analyses increased the summary outcomes for orbital invasion to 0.81 (0.64–0.91) for sensitivity, to 0.94 (0.89–0.97) for specificity, and to 0.96 (0.93–0.97) for NPV.

### 3.3. Quality Assessment

The patient selection domain always resulted in an “unclear” risk of bias, as it was not described in the studies, and the samples consisted of patients who had undergone tumor resection (Tables S2 and S3). The flow and timing were a source of “high bias”, while the interval between MRI and surgery was defined in only one study. In cases of rapidly growing tumors and to exclude discrepancies, an MRI is recommended to be performed the day before surgery [7]. Verification bias was identified in only two studies [7,15] that used two different reference standards (risk of bias) to confirm tumor invasion. Studies did not report a blinded review of the index test [16,26] (risk of bias) or the reference standard [6,7,14,15,16,26] (applicability issues). Various MRI criteria were used to assess the invasion of sinonasal tumors, but some studies [15,26] did not provide this information (Table S5).

**Figure 4:**
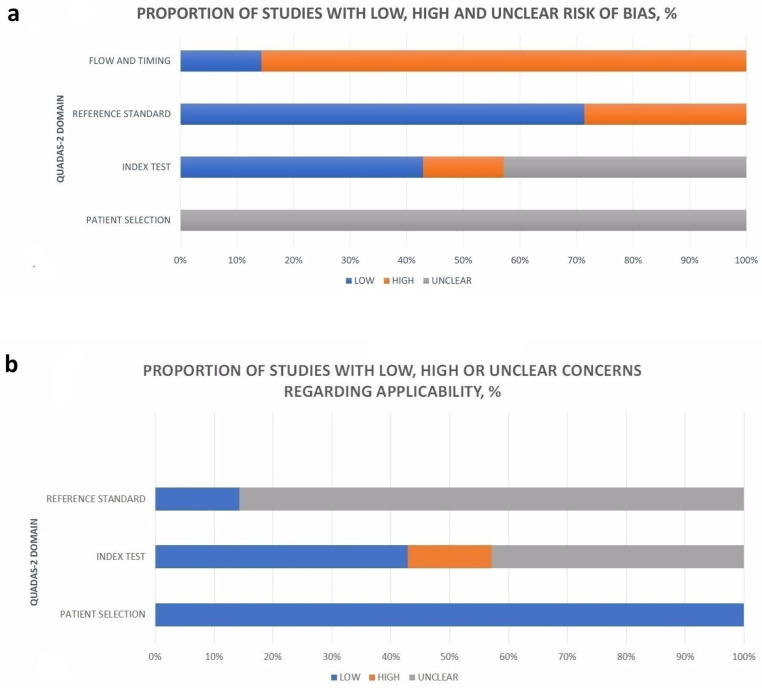
Graphical display of the risk of bias (**a**) and applicability concerns (**b**).

Studies were judged as “high risk of bias” when they presented high and/or unclear bias in ≤2 domains (Figure 4). An overall judgment relating to applicability concern was “low” for only one study [25]. Other studies were rated as “at risk of bias” for applicability, since they scored “high” or “unclear” in one [15,16] or two [6,7,14,26] domains.

### 3.4. Primary Tumor Histology and Location

Data on tumor histology were reported in all studies, but sinonasal tumor subsites were reported in only six [7,14,15,16,25,26] studies (Tables S6 and S7). The most common histological tumor types were olfactory neuroblastoma (ON), adenocarcinoma, and SCC. The ethmoid sinus was the most common tumor subsite with orbital and/or intracranial invasion.

### 3.5. MRI Technique and Protocols

A total of four (63%) studies [14,15,16,25] used 1.5 T MRI; one study also used 3 T [15]; and others (37%) did not report the field strength [6,7,26]. Four studies (63%) provided data on the MRI protocol [14,15,16,25] (Table S8). The main sequences for precontrast MRI were T2- and T1-weighted images without fat suppression (FS). For postcontrast MRI, axial and/or coronal T1-weighted FS images [15,16] were the most common (Table S9).

### 3.6. MRI Features of the Intracranial and Orbital Invasion of Sinonasal Tumors

Six studies [6,7,14,15,16,25] reported MRI features, and five [6,7,14,16,25] reported MRI criteria for orbital and/or intracranial invasion (Table S5), while others did not [15,26]. The most common MRI features of ICI were linear or nodular dural enhancement and dural thickening. Orbital invasion was recorded as invasion of the fat between the periorbita and extraocular muscles (EOM).

## 4. Discussion

### 4.1. Diagnostic Performance

We determined an overall accuracy of 0.80 for the ICI and 0.88 for orbital invasion of SNMs using MRI. MRI had higher overall accuracy, specificity, and NPV than ICI for orbital invasion. Evidence of significant heterogeneity across studies was associated more strongly with orbital invasion, the main reasons for which were the different invasion criteria applied (Table S5).

Meerwein et al. [15] assessed tumor invasion of the anterior skull base (ASB) and medial orbital wall (MOW) as a whole, without separately evaluating individual anatomical structures (the dura and periorbita). When assessing the diagnostic accuracy of MRI for orbital invasion, we included MRI data on stage B-F orbital involvement [16] alongside data on orbital content invasion [7].

Some studies were excluded for using inappropriate MRI criteria for assessing periorbital invasion [4,12] (Table S4). We did not evaluate brain invasion due to the limited number of patients in the studies [7,14,26].

Studies provided limited data on false imaging diagnoses (FP and FN) [7,15,16,25]. FP results were more common when assessing the dural and orbital bony wall [7,16] and MOW [15] invasion, whereas FNs were more common when assessing the bony skull base [7] and orbital fat involvement [13]. The rate of false results was equally high for ASB invasion [15].

The reported FP results were linked with discontinuous dural enhancement [27] and difficulty in the assessment of periorbital invasion due to peritumoral inflammation or erosion of the lamina papyracea by the growing tumor in the MOW [15].

The reported FN results were associated with the absence or misinterpretation of dural enhancement [6,25,28] due, respectively, to microscopic or focal dural invasion [28] with previous surgery or chemotherapy, which reduced accurate visualization of the extraconal fat and bony layer of the orbit [16].

Overall, the present study highlights the challenges when assessing intracranial and orbital invasion in SNMs using MRI. The high rate of FNs [6,13,15] and FPs [7,15,16] observed in this study for dural and orbital invasion cautions against relying solely on MRI when determining the optimal surgical approach [6,26,29].

### 4.2. Primary Tumor Histology and Location

Due to the small sample, two studies [14,25] could not be subjected to an analysis of the tumor’s histological type and location. The large sample with ON [26] influenced the frequency of histological types and the localization of primary tumors in general. SCC and adenocarcinoma [6], SCC [16], intestinal adenocarcinoma [7], and adenocarcinoma [15] were predominant among the histological types, which is in line with the findings of other studies [4,30,31] (Table S10). In the reviewed studies, the most common tumor subsites were the ethmoid sinus [7,26], maxillary sinus [16], and nasal cavity [15], which partially reflects the data from other studies [2,31] (Table S7).

Dural invasion was observed in 26% of cases of SNMs [7]. The incidence of invasion of the orbital bony wall was 20% [7]; that of the periorbita was 14.6% [16]; that of the orbital content was 8% [7]; and that of the MOW was 28.8% [15].

In conclusion, the data provided were limited to analyzing invasion incidence based on the histological type and location of SNMs.

### 4.3. MRI Technique and Protocols

Only Ferrari et al. [16] utilized a high-resolution MRI protocol to evaluate orbital invasion. Most authors [7,15,16] instead used axial, coronal, and sagittal [14] T2 images to assess orbital and intracranial invasion.

The importance of using T2-weighted sequences in assessing dural and orbital invasion was also reported [12,32], with the coronal view being superior for assessing the periorbita. Sagittal views could complement orbital floor and cribriform plate assessment [1,3].

In the reviewed studies, precontrast T1-WI was used in different projections and was considered sufficiently sensitive to evaluate bony orbit and periosteum and bony skull base invasion [14,33,34]. To distinguish tumor tissue from residual mucus in the adjacent sinuses, it is recommended to analyze pre- and postcontrast T1 and T2 sequences [1,35,36].

The studies preferred axial, coronal [7,15], and sagittal [16] contrast-enhanced T1-WI with FS for MRI analyses of intracranial and orbital tumor invasion, respectively. Postcontrast sagittal and coronal T1-weighted sequences allow differentiation of the bone–periosteum complex, dura, and subarachnoid space, as well as assessment of dural continuity and direct intracranial tumor extension [1,3,28,34,37]. On postcontrast T1-WI, the periorbita was less strongly enhanced than tumor tissue [12].

The use of an FS background on postcontrast FS sequences improves the detection of intracranial and orbital invasion [18,34]. FS postcontrast axial three-dimensional gradient echo T1 (VIBE) [16] and short tau inversion recovery (STIR) [32] sequences have also been used to improve the assessment of orbital fat invasion. STIR can be useful in overcoming skull base artifacts in FS sequences based on frequency-selective pulses [3]. Dedicated MRI protocols are recommended to improve the assessment of orbital invasion (coronal STIR, axial and coronal pre- and postcontrast T1, and a slice thickness of 3 mm) [38].

The studies utilized a range of MRI protocols and scanners. T1, T2, and postcontrast T1 with FS are the most appropriate MRI protocols for the intracranial and orbital invasion of SNMs. The periorbita and orbital invasion by tumors can be best assessed using coronal T2-WI.

### 4.4. MRI Features of Intracranial Invasion by Sinonasal Malignancies

The MRI features of ICI of SNMs with direct spread have been detailed in only a few studies identifying the predictors of dural invasion [6,7,25].

Across the studies, the types of ICI, such as bony skull base (SB), dura, and brain invasions, were investigated. Two studies described the MRI features of bony SB invasion, such as the loss of an asignal zone between the tumor and brain on T1WI [14] and minor/major modifications of the bone, the latter being a more reliable predictor of bony SB invasion (PPV 89%) [7].

On postcontrast MRI, signs of dural lesions were evaluated based on three key aspects: dural enhancement and thickening and loss of the hypointense zone (Table S5). The cut-off for dural thickness was 1 to ≥2 mm [6], >2 mm to ≤2 mm [7], and 5 mm [15,25]. Linear and nodular types of dural enhancement were also described; if both types were present, the enhancement was considered nodular [25].

Linear dural enhancement was found to occur in benign and malignant tumors and was not statistically associated with dural invasion but rather represented reactive changes [6,14,18,25,28,37]. Pial enhancement, along with other MRI features, was correlated with dural invasion [25] but remained very difficult to discriminate [18] and less sensitive for predicting dural invasion [8].

Dural invasion was observed in all malignant sinonasal tumors [6,25]. The reported predictors of dural invasion on postcontrast MRI were nodular dural enhancement [6,7,25]; dural thickening of >5 mm [25], ≥2 mm [6], and >2 mm [7]; loss of the hypointense zone [6,28,37]; and irregular deformation of the dura and a contact angle over 45° between the tumor and dura [7]. The absence of nodular dural enhancement did not exclude dural invasion (*p* = 0.055) [6].

The studies found a moderate-to-strong statistical correlation between dural invasion and nodular dural thickening of >5 mm (*p* < 0.001) [25] and >2 mm (PPV 87%) [7], linear thickening of ≥2 mm (*p* = 0.042) [6], and loss of the hypointense zone (*p* < 0.001) [6,28,37], the latter representing the space between the enhancing tumor and the reactively changed dura.

Irregular deformation of the dura, a contact angle over 45° between the tumor and dura, and nodular dural enhancement of >2 mm (Table S5) were found to predict dural invasion in a large study on SNMs (PPVs > 85%). However, the MRI finding of a “contact angle over 45° between the tumor and dura” was not discussed in other studies [7].

According to McIntyre et al. [6], nodular dural enhancement (PPV, 100%, *p* = 0.055), dural thickening of ≥2 mm (PPV, 100%, *p* = 0.042), and loss of the hypointense zone (PPV, 92.3, *p* < 0.001) on postcontrast MRI are the predictors of dural invasion in SNMs, the latter being the most reliable predictor.

Dural involvement immediately upstages patients as T4a/b and is critical for determining the extent of resection [8,39]. Linear dural enhancement can be seen with both benign and malignant tumors, while dural invasion is more specific for malignant sinonasal tumors. Nodular dural enhancement, dural thickening of ≥2 mm, and loss of the hypointense zone on postcontrast MRI are the predictors of dural invasion, the latter being the most reliable predictor in malignant tumors. However, the absence of MRI features of dural invasion cannot exclude tumor involvement.

### 4.5. MRI Features of Orbital Invasion by Sinonasal Malignancies

Orbital invasion in various tumors has been investigated in a limited number of studies [7,12,13,15,16], only three of which [7,15,16] met the inclusion criteria in our study (Table 1). In the reviewed studies, the orbital bony wall, periorbita, extraconal fat, nasolacrimal system, and orbital content were assessed separately [7,12,13,16], or as a single structure [15], using various MRI criteria (Table S5).

For the assessment of orbital bony wall invasion, the MRI feature of “major (≥2 mm) modification” yielded a low PPV (47.5%) [7], while “partial interruption of the periorbita” yielded a high PPV (87.5%) and diagnostic accuracy (84.8%) [16]. Disruption of the orbital bony wall did not indicate orbital invasion [40].

The periorbita was found to be hypointense and isointense relative to the EOM on T1 and T2-WI [12]. This feature is usually indistinguishable from the bony wall [13,39,40]. However, Kim et al. identified two separate periorbital layers (the outer bony wall and the inner periorbita) on the coronal T2 images of six patients. The periorbita exhibited less contrast enhancement on postcontrast images than the tumor [12] and is considered to be a prognostic barrier for orbital invasion [16].

The MRI sign “tumor abutting periorbita” did not correlate with periorbital invasion [12]. Periorbital infiltration leads to disruption of the periorbita’s hypointense MR signal and a loss of clear margins [1,34,36].

Infiltration of the extraconal fat between the periorbita and EOM on MRI has been described as a predictor of orbital invasion and as a critical structure in the decision to preserve the orbit [12,13,15,16,41], albeit with a variable PPV: 33.3% [7], 80% [13], and 70.0% [16]. When assessing orbital invasion, MRI stages A, B, and C represented a tumor invading the extraconal fat and/or medial part of the lacrimal sac with preservation of the EOM (PPV 77.1%, diagnostic accuracy 71.6%) [16]. The diagnostic accuracy for the assessment of nasolacrimal system invasion was 89% [13].

Orbital invasion might be accompanied by periorbital disruption or thickening, the latter representing reactive changes to the tumor, with an average periorbital thickness of 1.2 mm [12]. Orbital invasion was not considered in instances where the periorbita was intact [13].

In another study [13], none of the eleven MRI features of orbital invasion demonstrated a diagnostic accuracy of >79% and were not statistically correlated with orbital invasion. The EOM (100%) and orbital fat invasion (80%) were the most specific features of orbital invasion but presented low sensitivity (<11 and 40%, respectively). Tumors adjacent to the periorbita were the most sensitive (90%) but also offered low specificity (29%).

Meerwein et al. assessed “medial orbital wall” invasion in a large sample of SNMs; the diagnostic accuracy of the MRI ranged from 89.0% for Reader 1 to 93.2% for Reader 2. Due to the high number of FPs, NPV was higher than PPV in both cases. In a study by Salfrant et al., the MRI features of orbital content invasion presented low PPVs (<50%). Radiology obtained low sensitivity (28.6%) and a high NPV (93.4%) [7]. Intraconal compartment invasion (MRI stages D, E, and F) was an indication for orbital ablation and yielded a diagnostic accuracy of <80.0% [16].

A recent publication showed that pretreatment MRI staging could improve the assessment of orbital invasion [16], better depicting the relationship of the tumor with the anatomical structures of the orbit (orbital wall, extraconal fat, and EOM) than prior studies [7,13,15], making orbital-sparing surgery possible for many patients. In MRI stages C or D (extraconal fat and/or EOM invasion with no clinical symptoms of EOM involvement), the decision to perform orbital ablation was made intraoperatively using frozen sections [16].

Infiltration of the extraconal fat beyond the periorbita is an MRI feature of orbital invasion. Studies have used varying MRI features and criteria, making overall synthesis difficult to achieve. Therefore, the diagnostic accuracy of MRI varies between studies.

### 4.6. Future Directions

This systematic review and meta-analysis highlights the need to conduct more prospective studies on larger samples using MRI to objectively assess the diagnostic accuracy of MRI sequences and predictors for orbital and intracranial invasion and obtain a reliable estimate of specificity and sensitivity. Further studies should include detailed information on MRI vendors, imaging techniques and protocols, and actual rates of MRI false diagnoses to obtain more accurate scientific data.

## 5. Limitations

The limitations of this review include a small number of predominantly retrospective studies that were examined, some with a small subset of patients [14,25]. Moreover, the search for studies was limited to research published in English only, which represents an additional limitation. These studies also provided limited data on false imaging diagnoses (FP and FN) [7,15,16,25].

Due to the small sample, two studies [14,25] could not be analyzed regarding the tumor’s histological type and location. The data provided were limited to analyzing invasion incidence based on the histological type and location of SNMs.

Moreover, the reviewed studies used different MRI scanners, protocols, reference methods, MRI features, and criteria for tumor invasion, sometimes with a lack of relevant data. In these cases, we selected the most appropriate MRI invasion criteria to evaluate dural or orbital invasion. We had to exclude or extract incomplete data from studies with a heterogeneous sample of patients that did not separately provide data on SNMs. These decisions may have introduced bias into our study.

## 6. Conclusions

Accurately detecting sinonasal cancer invasion of the dura and orbit is crucial to determining the need for craniofacial surgery. MRI yields moderate-to-high diagnostic accuracy for intracranial and orbital invasion but suffers from limitations leading to false diagnoses. A literature review confirmed that the most common histological tumor types with orbital and/or cranial invasion are ON, adenocarcinoma, and SCC, with the most common tumor subsite being the ethmoid sinus. The presence of a non-negligible rate of FNs and FPs in dural and orbital invasion cautions against relying solely on MRI when determining the optimal surgical approach. In addition, dural invasion is more specific for malignant sinonasal tumors and can be predicted from a loss of the hypointense zone on postcontrast MRI. Infiltration of the extraconal fat beyond the periorbita is an MRI feature of orbital invasion to consider.

## Figures and Tables

**Table 1:** Summary of studies included in the systematic review and meta-analysis.

Authors	Year	Design	N of Patients with Invasion	Mean Age	TP	TN	FP	FN	Se	Sp	PPV	NPV	Acc
**Studies examining intracranial invasion**													
McIntyre J.B. et al. [6]	2012	prosp.	17	54.6	13	1	2	1	0.93	0.33	0.87	0.50	0.82
Salfrant M. et al. [7]	2021	retr.	160	57	33	97	21	9	0.79	0.82	0.61	0.92	0.81
Meerwein C.M. et al. [15]	2020	retr.		67									
Reader 1			73		18	42	2	11	0.62	0.96	0.90	0.79	0.82
Reader 2			73		23	33	11	6	0.79	0.75	0.68	0.85	0.77
Eisen M.D. et al. [25]	1996	retr.	4	53	2	1	-	1	0.67	1.00	1.00	0.50	0.75
Geltzeiler M. et al. [26]	2023	retr.	166	52.2	73	57	17	19	0.79	0.77	0.81	0.75	0.78
Ishida H. et al. * [14]	2002	retr.	3	47.3	1	2	-	-	1.00	1.00	1.00	1.00	1.00
**Studies examining orbital invasion**													
Salfrant M. et al. [7]	2021	retr.	160	57	4	142	4	10	0.29	0.97	0.50	0.93	0.91
Ferrari M. et al. [16]	2021	retr.	123	64	60	25	10	28	0.68	0.71	0.86	0.47	0.69
Meerwein C.M. et al. [15]	2020	retr.		67									
Reader 1			73		12	54	5	2	0.86	0.92	0.71	0.96	0.90
Reader 2			73		19	49	3	2	0.91	0.94	0.86	0.96	0.93

TP—true positive; TN—true negative; FP—false positive; FN—false negative; Se—sensitivity; Sp—specificity; PPV—positive predictive value; NPV—negative predictive value; Acc—diagnostic accuracy; Studies examining intracranial invasion—include studies evaluating the intracranial invasion of sinonasal malignancies; Studies examining orbital invasion—include studies evaluating the orbital invasion of sinonasal malignancies; prosp.—prospective; retr.—retrospective; Ishida H. et al. *—this study was included only in the systematic review and was not included in the meta-analysis.

## Data Availability

No new data were created or analyzed in this study. Data sharing is not applicable to this article.

## Supplementary Materials

The following supporting information can be downloaded at: https://www.mdpi.com/article/10.3390/jcm13247556/s1, Checklist S1: PRISMA 2020 Checklist; Table S1: Database search terms and strategy; Table S2: QUADAS-2 assessments; Table S3: QUADAS-2 domains and questions; Table S4: Summary of the reasons for excluding studies after screening for eligibility; Table S5: MRI features and invasion criteria for orbital and intracranial invasion of the sinonasal malignancies; Table S6: Summary of tumors evaluated for orbital and/or intracranial extension; Table S7: Summary of the sinonasal tumors’ subsites with orbital and/or intracranial invasion; Table S8: Technical details of MRI vendors and MRI protocols in the studies; Table S9: MRI protocols used in the studies; Table S10: Summary of the histological forms of sinonasal tumors in the studies.
