# Supplementary material for: Diagnostic Accuracy of MRI for Orbital and Intracranial Invasion of Sinonasal Malignancies: A Systematic Review and Meta-Analysis
Source: J Clin Med. 2024 Dec 12;13(24):7556. doi: 10.3390/jcm13247556 (PMC11728326; doi:10.3390/jcm13247556)
Supplement: Supplementary file 1 [file jcm-13-07556-s001.zip › jcm-3315757-supplementary.pdf]

Journal of
Clinical Medicine

MDPI

Systematic Review

Diagnostic accuracy of MRI for orbital and intracranial invasion of sinonasal malignancies: a systematic review and meta-analysis

Umida Abdullaeva[1]*, Bernd Pape [2,3], Jussi Hirvonen[4]

[1] Department of Radiology, Tashkent City Branch of the Republican Specialized Scientific and Practical Medical Center of Oncology and Radiology, 100054 Tashkent, Uzbekistan; umidasamira2@gmail.com
[2] Department of Biostatistics, University of Turku and Turku University Hospital, 20521 Turku, Finland; bernd.pape@tyks.fi
[3] School of Technology and Innovations, University of Vaasa, 65101 Vaasa, Finland; bernd.pape@uwasa.fi
[4] Department of Radiology, Faculty of Medicine and Health Technology, Tampere University Hospital and Tampere University, 33520 Tampere, Finland; jussi.hirvonen@utu.fi
* Correspondence: umidasamira2@gmail.com; Tel.: (+998)-90-958-94-54

Table S1. Database search terms and strategy.

Key words and operators	Combination of key words
1 sinonasal neoplasm	
2 MRI	
3 intracranial extension	
4 invasion	
5 paranasal sinus cancer	
6 extension	
7 maxillary sinus neoplasm	
8 involvement	
9 orbital invasion	
10 orbital extension	
11 orbital involvement	
12 cranial invasion	
13 intracranial invasion	
14 paranasal sinus neoplasms	
15 paranasal neoplasm	
16 intracranial involvement	
17 cranial extension	
18 nasal cavity tumors	
19 nasosinusal neoplasms	
20 nasal neoplasm	
21 nasal cancer	
22 nose neoplasm	
23 ethmoidal tumors	
24 sphenoidal tumors	
25 sphenoidal neoplasm	
26 neoplasm	
27 tumor	
AND/1,2	sinonasal neoplasm AND MRI

AND/1,4	sinonasal neoplasm AND invasion
AND/1,6	sinonasal neoplasm AND extension
AND/1,8	sinonasal neoplasm AND involvement
AND/1,2,4	((sinonasal neoplasm) AND (MRI)) AND (invasion)
AND/1 ,2,9	((sinonasal neoplasm) AND (MRI)) AND (orbital invasion)
AND/1 ,2,10	((sinonasal neoplasm) AND (MRI)) AND (orbital extension)
AND/1 ,2,11	((sinonasal neoplasm) AND (MRI)) AND (orbital involvement)
AND/1 ,2,12	((sinonasal neoplasm) AND (MRI)) AND (cranial invasion)
AND/1 ,2,13	((sinonasal neoplasm) AND (MRI)) AND (intracranial invasion)
AND/OR (15 OR 14) AND 2 (11 OR 9 OR 10)	(paranasal neoplasm OR paranasal sinus neoplasms) AND MRI AND (orbital involvement OR orbital invasion OR orbital extension)
AND/OR (15 OR 14) AND 2 (13 OR 12 OR 16)	(paranasal neoplasm OR paranasal sinus neoplasms) AND MRI AND (intracranial invasion OR cranial extension OR intracranial involvement)
AND/OR (15 OR 1) AND 2 (13 OR 12 OR 16)	(paranasal neoplasm OR sinonasal neoplasm) AND MRI AND (intracranial invasion OR cranial extension OR intracranial involvement)
AND/OR (5) AND 2 (4 OR 10 OR 16)	(paranasal sinus cancer) AND MRI AND (invasion OR orbital extension OR intracranial involvement)
AND/OR (7OR18) AND 2 (9 OR 10 OR 11)	(maxillary sinus neoplasm OR nasal cavity tumors) AND MRI AND (orbital invasion OR orbital extension OR orbital involvement)
AND/OR (7OR18) AND 2 (12 OR 17 OR 13)	(maxillary sinus neoplasm OR nasal cavity tumors) AND MRI AND (cranial invasion OR cranial extension OR intracranial involvement)
AND/19,2	nasosinusal neoplasms AND MRI
AND/20,2	nasal neoplasm AND MRI
AND/OR 21,4,22,9	nasal cancer AND invasion OR nose neoplasm AND orbital invasion
AND/OR 21,12,22,11	nasal cancer AND cranial invasion OR nose neoplasm AND orbital involvement
AND/OR (23OR24) AND 2 (12 OR 17 OR 13)	(ethmoidal tumors OR sphenoidal tumors) AND MRI AND (cranial invasion OR cranial extension OR intracranial involvement)
AND/OR (23OR24) AND 2 (9 OR 10 OR 11)	(ethmoidal tumors OR sphenoidal tumors) AND MRI AND (orbital invasion OR orbital extension OR orbital involvement)

AND/OR (1OR5) AND (26 OR 27) AND 2 (4 OR 5 OR 8)	(sinonasal OR paranasal) AND (neoplasm or tumor) AND MRI AND (invasion OR extension OR involvement)

Table S2. QUADAS-2 assessments.

	Risk of bias				Applicability concern		
**First authors**	**Patient selection**	**Index test**	**Reference standard**	**Flow and timing**	**Patient selection**	**Index test**	**Reference standard**
McIntyre J.B. et al.[6]	unclear	low	low	high	low	unclear	unclear
Salfrant M. et al.[7]	unclear	low	high	low	low	unclear	unclear
Ferrari M. et al.[16]	unclear	unclear	low	high	low	low	unclear
Meerwein C.M. et al.[15]	unclear	unclear	high	high	low	low	unclear
Eisen M.D. et al.[25]	unclear	low	low	high	low	low	low
Geltzeiler M. et al.[26]	unclear	high	low	high	low	high	unclear
Ishida H. et al.[14]	unclear	unclear	low	high	low	unclear	unclear

Table S3. QUADAS-2 domains and questions

1	**RISK OF BIAS**<br>Risk of bias is judged to be "low", "high", or "unclear". If the answers to all signaling questions for a domain are "yes", then the risk of bias can be judged low. If any signaling question is answered "no", the potential for bias exists. The "unclear" category should be used only when insufficient data are reported to permit a judgment.
1a	***Patient selection***
	**Could the selection of patients have introduced bias? (Low/High/Unclear risk)**
	*Was the selection method of patients reported?
	*Was a random or consecutive sample enrolled?
	* Did the study have inappropriate exclusions?
	Random—low bias, consecutive—low bias, no information –
	Random/consecutive—low bias, no information—unclear bias, studies enrolled patients with known disease—high bias
1b	***Index test***

	Could the conduct or interpretation of the index test have introduced bias? (Low/High/Unclear risk)
	*Were all radiologists blinded to the results of the reference standard during MRI reporting?
	*Did the study report the MRI criteria for invasion?
	Blinded review and/or study reported MRI criteria for invasion—l**ow bias**, only one of previous questions—**unclear bias,** no information regarding both questions**—high bias**
1c	***Reference standard***
	Could the reference standard or its conduct or interpretation have introduced bias? (Low/High/Unclear risk)
	*Which reference method was used?
	*Did all patients undergo the same reference method?
	Histology and surgery used—l**ow bias**, only surgery used—**unclear bias**, different reference methods**—high bias**
1d	***Flow and timing***
	Could the patient flow have introduced bias?
	Time between index test and surgery reported—low bias, no data reported**—high bias**
2	**APPLICABILITY CONCERN**<br>Concerns regarding applicability were rated as “low”, “high”, or “unclear”. The “unclear” category was used only when insufficient data were reported.
2a	***Patient selection***
	Are there concerns that the included patients (tumors) do not match the review question?
	*Was tumor histology reported?
	Reported—low bias, not reported—high bias
2b	***Index test***
	Are there concerns that the index test or its conduct or interpretation differs from the review question? (**Low/High/Unclear risk**)
	*Did all studies report data on MRI protocols and vendors?
	*Did all studies provide data regarding the number and experience of the study radiologists?
	MRI protocol and number of radiologists reported—low bias, only criteria reported—**unclear**, no data reported**—high bias**
2c	***Reference standard***
	*Was the result of the reference standard interpreted without prior knowledge of the index test?—Low risk for all studies
	Blinded review—low risk, data not provided—unclear bias

Table S4. Summary of the reasons for excluding studies after screening for eligibility.

№	First authors	Year	Causes	DOI
1	Mario Turri-Zanoni et al.	2019	No MRI data for the table 2x2	doi.org/10.1002/hed.25759

2	Carlos Suarez et al.	2003	No MRI data for the table 2x2	doi.org/10.1002/hed.10358
3	Valerie J. Lund et al.	1996	No MRI data for the table 2x2	doi.org/10.1097/00005537-199605000-00007
4	C D Phillips et al.	1997	No MRI data for the table 2x2	doi.org/10.1148/radiology.202.2.9015077
5	S.-W. Park et al.	2009	No reference test	doi.org/10.3174/ajnr.A1317
6	Hilary S. Serracino et al.	2013	No MRI data for the table 2x2	doi.org/10.1097/NEN.0b013e318299c40f
7	Wang X. et al.	2014	Tumor invasion was not evaluated	-
8	Yan, Zhongyu et al.	2014	Tumor invasion was not evaluated	10.1097/RCT.0b013e3182a7764c
9	Mohammed A. Gomaa et al.	2013	No reference test	doi.org/10.4137/CMENT.S10678
10	Cuiping Zhou et al.	2015	Tumor invasion was not evaluated	DOI: 10.1016/j.clinimag.2014.08.004
11	Xinyan Wang et al.	2016	No reference test	doi.org/10.1002/jmri.25484
12	Kerem Ozturk et al.	2019	No reference test	doi.org/10.1177/1971400919873895
13	Carol H et al.	2018	Tumor invasion was not evaluated	doi.org/10.1002/lary.27582
14	Junjie Zeng et al.	2021	No reference test	doi.org/10.1259/dmfr.20210030
15	N. Lin et al.	2020	Tumor invasion was not evaluated	doi.org/10.1016/j.crad.2020.08.007
16	Yun Chen et al.	2020	No reference test	doi.org/10.1007/s00234-020-02471-3
17	Zheng Li et al.	2020	Tumor invasion was not evaluated	doi.org/10.1007/s00330-020-06838-1
18	Jun-hua Liu et al.	2023	No reference test	doi.org/10.1186/s12880-023-01062-x
19	Hangzhi Liu et al.	2023	Tumor invasion was not evaluated	doi.org/10.1007/s00234-023-03164-3
20	D.R. Shatzkes et al.	2016	Tumor invasion was not evaluated	doi.org/10.3174/ajnr.A4841
21	Akira Baba et al.	2022	Tumor invasion was not evaluated	DOI: 10.1007/s00234-022-03009-5
22	Yue-fei Deng et al.	2016	No separate MRI data for the table 2x2	doi.org/10.1007/s00405-016-4003-8
23	Dennis H. Kraus et al.	1992	Not all patients had the reference method; no separate data in groups with and without the reference standard	doi.org/10.1288/00005537-199206000-00006
24	Qingqiang Zhu et al.	2015	No reference test	doi.org/10.1186/s12957-015-0475-z
25	Hiroki Kato et al.	2013	No reference test	doi.org/10.1007/s11604-013-0247-z

26	Kenichiro Iwami et al.	2020	No MRI data for the table 2x2	10.1055/s-0039-3402034
27	D Pickuth et al.	1999	No separate CT and MRI data	10.1259/bjr.72.863.10700820
28	Keita Oikawa et al.	2007	Tumor invasion was not evaluated	10.1177/000348940711600909
29	Ricardo L. Carrau et al.	2009	No imaging data	doi.org/10.1097/00005537-199902000-00012
30	Tian Yu,Yi-Kai Xu et al.	2009	No reference test	10.1007/s00234-009-0581-0
31	Tassel, Pamela Van et al.	1991	No reference test	doi.org/10.1097/00004728-199105000-00008
32	Som P.M. et al.	1993	Tumor invasion was not evaluated	PMC8332439
33	Hye Yeon Choi et al.	2019	No MRI data for the table 2x2; heterogeneous sample	10.1177/0284185118778883
34	G. Mortuaire et al.	2017	No separate CT and MRI data for the table 2x2	doi.org/10.1111/coa.12827
35	Amar Miglani et al.	2019	No MRI data for the table 2x2	doi.org/10.1002/lio2.305
36	M. G. M. Hunink et al.	1990	No MRI data for the table 2x2	10.1007/BF00589116
37	Mafee MF et al.	1993	No MRI data for the table 2x2	-
38	Dijana Podoreski et al.	2010	Various types of maxillofacial tumors; no separate data for sinonasal malignancies; reference standard uncertain	pubmed.ncbi.nlm.nih.gov/20635582/
39	Cruz, Antonio A. V. et al.	2014	No reference standard	10.1097/IOP.0b013e3182a7500e
40	Alessandro Vinciguerra et al.	2023	IC or orbital invasion was not assessed on MRI	doi.org/10.1002/alr.23057
41	H D Curtin et al.	1998	Review study	doi.org/10.1016/s0033-8389(05)70240-2
42	Marc D. Eisen BA et al.	2000	Sample consisted of benign and malignant tumors with different localizations; no separate data for different groups	doi.org/10.1002/1097-0347(200008)22:5%3C456::aid-hed3%3E3.0.co;2-n
43	R Tiwari et al.	1998	No MRI data for the table 2x2	https://pubmed.ncbi.nlm.nih.gov/9570623/
44	C Li. et al.	1993	Sample consisted of tumors with different localizations; not all patients had MRI with contrast enhancement	https://www.ncbi.nlm.nih.gov/pmc/articles/PMC8332772/

45	Zhenchao Sun et al.	2023	Invasion of extraocular muscles was evaluated; no periorbital invasion assessment	https://doi.org/10.1007/s00405-023-07874-5
46	D J Lee et al.	2014	No MRI data for the table 2x2	10.1016/j.ijom.2013.11.017
47	Hak Jin Kim et al.	2006	No MRI data for the table 2x2; inappropriate MRI criteria for assessing periorbital invasion	https://doi.org/10.2500/ajr.2006.20.2889
48	Aliasgar V Moiyadi et al.	2013	Sample consisted of skull base tumors with different localizations	https://pubmed.ncbi.nlm.nih.gov/24015414/
49	Markose S. et al.	2013	Case report	10.18502/cjn.v22i3.13802
50	Wu K. et al.	2020	IC or orbital invasion was not assessed on MRI	10.1080/01676830.2020.1852262
51	Melder K. et al.	2021	No MRI data for the table 2x2; conference abstract	10.1055/s-0041-1725349
52	Davis R.J. et al.	2018	Case report	10.1001/jamaoto.2017.3446
53	Gompel J.J.V. et al.	2018	No MRI data for the table 2x2	10.1055/s-0037-1606307
54	Ogawa T. et al.	2017	IC or orbital invasion was not assessed on MRI	10.5981/jjhnc.43.409
55	Lisan Q. et al.	2016	IC or orbital invasion was not assessed on MRI	10.1002/hed.24490
56	Batra P.S. et al.	2014	No MRI data for the table 2x2; conference abstract	10.1055/s-0032-1312158
57	Derdeyn C.P.	1994	No reference test	-
58	Hung Y. et al.	2018	Case report	-
59	Ahmadi J. et al.	1993	Sample consisted of tumors with different localizations; no separate MRI data from sinonasal malignant tumors	DOI: 10.1148/radiology.188.3.8351343
60	Ahmadi J. et al.	1994	Sample consisted of tumors with different localizations; no separate MRI data from sinonasal malignant tumors	DOI: 10.1227/00006123-199409000-00003
61	Wilms G. et al.	1991	Dural invasion was evaluated only for intracranial tumors	PMCID: PMC8331606
62	Hedyeh Ziai et al.	2018	IC or orbital invasion was not assessed on MRI	DOI: 10.1055/s-0037-1612617
63	W S McCary et al.	1996	IC or orbital invasion was not assessed on MRI	DOI: 10.1001/archotol.1996.01890180063015

64	Mohammed A Gomaa et al.	2013	IC or orbital invasion was not assessed on MRI	DOI: 10.4137/CMENT.S10678
65	D J Lee et al.	2014	No MRI data for the table 2x2	DOI: 10.1016/j.ijom.2013.11.017
66	Arana et al.	2004	Calvarial benign and malignant lesions were evaluated; no sinonasal tumors	DOI: 10.1007/s00234-004-1284-1
72	Connor S.E.J.	2015	Review	10.1016/j.nic.2015.07.007
76	Davis R.J. et al.	2018	Case report	10.1001/jamaoto.2017.3446
77	Ben Simon GJ et al.	2005	Orbital tumors were evaluated	10.1016/j.ophtha.2005.09.013
78	O. Chambres	2005	Only in French	10.1016/s0003-438x(05)82315-1
79	R Maroldi et al.	1996	Only in Italian	8628933
80	Eisen MD et al.	2000	Sample consisted of tumors with different localizations; no separate MRI data from sinonasal malignant tumors	10.1002/1097-0347(200008)22:5<456::aid-hed3>3.0.co;2-n
81	L. Zhang et al.	2020	IC or orbital invasion was not assessed on MRI	https://doi.org/10.4193/rhin19.240
82	R Maroldi et al.	2020	Book chapter	https://doi.org/10.1159/000457923
83	Zlochower, Avraham B. et al.	2021	Review	10.1097/RMR.0000000000000289
84	Famuyide, Akinrinola et al.	2021	Review	10.1097/RMR.0000000000000288

Notes:
IC—intracranial

Table S5. MRI features and invasion criteria for orbital and intracranial invasion of sinonasal malignancies.

N	Authors	The anatomic structure (unit) evaluated on MRI	MRI features	MRI invasion criteria
		**Studies evaluating intracranial invasion**		
1	McIntyre J.B. et al.[6]	**Dural invasion**	Dural enhancement (linear or nodular);<br>Presence or absence of the hypointense zone;<br>Dural thickening (mm)	≥2 mm of dural thickening, loss of hypointense zone, and nodular dural enhancement on postcontrast MRI are highly predictive of dural invasion by malignant sinonasal tumors
2	Salfrant M. et al.[7]	**Dural invasion**	**Bony skull base:**<br>Minor (<2 mm) modification;<br>Major (≥2 mm) modification;<br>**Dura free:**	Contact angle over 45° between the tumor and dura, irregular deformation of the dura adjacent to tumor, and nodular dural enhancement over 2

			Linear enhancement: ≤2 mm or >2 mm;<br>Nodular enhancement: ≤2 mm or >2 mm;<br>**Dura invaded:**<br>Smooth or irregular deformation;<br>Contact angle: ≤45◦ or >45◦	mm in thickness = MRI signs of dural invasion with the highest PPV
3	Meerwein C.M. et al.[15]	**Anterior skull base**	a) Infiltration of the bony cribriform plate/ periosteum,<br>(b) Dural thickening up to 5 mm,<br>(c) Nodular dural thickening or dural thickening >5 mm,<br>(d) Direct invasion of brain parenchyma	-
4	Eisen M.D. et al. [25]	**Dural invasion**	Pial, dural, linear, and nodular enhancement and the width of dural thickening	Pial enhancement, focal dural nodules, or dural thickening of more than 5 mm is highly accurate in predicting dural invasion
5	Geltzeiler M. et al.[26]	**Dural invasion**	-	-
6	Ishida H. et al.[14]	**Invasion of bony skull base and dura mater**	Asignal zone between the tumor and brain;<br>Enhanced linear shadow between the tumor and brain on Gd-enhanced MRI;<br>Intracranial mass	Hypertrophic linear shadow on Gd-enhanced MRI or thickened dura mater adjacent to the tumor represented **dural invasion**
		**Studies evaluating orbital invasion**		
1	Salfrant M. et al.[7]	**Orbital invasion**	**Orbital bony walls:**<br>Minor (<2 mm) modification;<br>Major (≥2 mm) modification;<br>**Orbital content free:**<br>Invasion of the fat between the tumor and oculomotor muscle;<br>Smooth or irregular deformation;<br>**Orbital content invaded:**<br>Contact angle: ≤45◦ or >45◦;<br>Invasion of the oculomotor muscle	No radiological sign had a significant predictive value; signs of orbital invasion had low PPVs (<50%)
2	Ferrari M. et al.[16]	**Orbital invasion**	MRI staging of orbital invasion:<br>**Stage A:** Tumor abutting the orbit, but the layer composed of orbital bony walls and periorbita was not transgressed;<br>**Stage B:** The layer composed of orbital bony walls and periorbita was partially interrupted, with no evidence of tumor within the extraconal fat or lacrimal sac;<br>**Stage C:** Tumor was identified within the extraconal fat and/or the medial portion of the lacrimal sac, with no	**Orbital wall invasio**n (stage A and B);<br>**Periorbital invasion** (stage A, B, and C);<br>**Extraconal fat invasion** (stage B, C, and D);<br>**Extrinsic ocular muscle invasion** (stage C, D, E, and F);<br>**Intraconal compartment invasion** (stage D, E, and F)

			signs suspicious of involvement of extrinsic ocular muscles;<br>**Stage D:** Tumor was identified up to the lateral portion of the lacrimal sac, and/or extrinsic muscle invasion was suspected based on the presence of tumor signal within the muscle(s), muscular enlargement, and/or abnormal muscular enhancement. No extension to the intraconal compartment was suspected;<br>**Stage E:** Tumor was identified within the intraconal compartment, defined as the space surrounded by the inner aspect of rectus muscles and/or into the preseptal space; **Stage F:** Tumor was identified within the orbital apex, which was defined as all the orbital content (excluding the bony walls) located posterior to the paracoronal plane passing through the posterior ethmoidal foramen and the lateral end of the superior orbital fissure	
3	Meerwein C.M. et al.[15]	**Medial orbital wall**	(a) Bony orbit and periorbita, (b) Extraconal adipose tissue, (c) Extraocular eye muscles, (d) Intraconal structure infiltration	-

Table S6. Summary of the tumors evaluated for orbital and/or intracranial extension.

№	Authors	Tumor histology	Site of tumor origin
1	McIntyre J.B. et al.[6]	Adenocarcinoma (9)<br>SCC (9)<br>Mucosal melanoma (4)<br>Olfactory neuroblastoma (4)<br>ACC (3)<br>SNUC (3)<br>Clear-cell carcinoma (1)<br>Chondrosarcoma (1)<br>Chordoma (1)<br>Osteosarcoma (1)<br>Metastatic-renal-cell carcinoma (1)<br>Sarcoma (1)<br>Spindle-cell carcinoma (1)	Sinonasal
2	Salfrant M. et al.[7]	Intestinal-type adenocarcinoma 72 (41%)<br>Esthesioneuroblastoma 36 (20%)<br>Non-intestinal-type adenocarcinoma 28 (16%)<br>SCC 10 (6%)<br>Mucosal melanoma 9 (5%)<br>Neuroendocrine carcinoma 6 (3%)<br>Rhabdomyosarcoma 3 (2%)	Ethmoid 155 (88%)<br>Olfactory cleft 13 (7%)<br>Nasal septum 3 (2%)<br>Middle turbinate 1<br>Frontal 1<br>Maxillary bone 1<br>Orbit 1

		Other 12	Sphenoid 1
3	Ferrari M. et al.[16]	**Squamous-cell carcinoma:** 43/81 (53.1%)<br>o Keratinizing, classic variant: 21/43 (48.8%)<br>o Non-keratinizing: 7/43 (16.3%)<br>o Ex-inverted papilloma: 6/43 (14.0%)<br>o Spindle-cell: 4/43 (9.3%)<br>o Adenosquamous carcinoma: 3/43 (7.0%)<br>o Adenoid: 1/43 (2.3%)<br>o Basaloid: 1/43 (2.3%);<br>**Sinonasal adenocarcinoma**: 20/81 (24.7%)<br>o Intestinal-type adenocarcinoma: 19/20 (95.0%)<br>o Non-intestinal-type adenocarcinoma: 1/20 (5.0%);<br>**Small-cell neuroendocrine carcinoma**: 5/81 (6.2%)<br>**Sinonasal undifferentiated carcinoma**: 4/81 (4.9%)<br>**Carcinoma NOS**: 3/81 (3.7%)<br>**Basal-cell carcinoma**: 2/81 (2.5%)<br>**INI-1-deficient carcinoma**: 2/81 (2.5%);<br>**Minor salivary gland carcinomas**: 18/123 (14.6%)<br>· Adenoid cystic carcinoma: 14/18 (77.8%)<br>· Epithelial-myoepithelial carcinoma: 1/18 (5.6%)<br>· Myoepithelial carcinoma: 1/18 (5.6%)<br>· Salivary duct carcinoma: 1/18 (5.6%)<br>· Polymorphous adenocarcinoma: 1/18 (5.6%);<br>**Neuroectodermal tumors:** 15/123 (12.2%)<br>· Mucosal melanoma: 10/15 (66.7%)<br>· Olfactory neuroblastoma: 5/15 (33.3%);<br>**Mesenchymal tumors**: 7/123 (5.7%)<br>· Undifferentiated pleomorphic sarcoma: 2/7 (28.6%)<br>· Chondrosarcoma: 1/7 (14.3%)<br>· Extrapleural solitary fibrous tumor: 1/7 (14.3%)<br>· Fibrosarcoma: 1/7 (14.3%)<br>· Myofibroblastic sarcoma: 1/7 (14.3%)<br>· Myxofibrosarcoma: 1/7 (14.3%);<br>**Embryonal tumors** (teratocarcinosarcoma): 2/123 (1.6%)	Nasoethmoidal: 58/123 (47.2%)<br>Maxillary: 65/123 (52.8%)
4	Meerwein C.M. et al.[15]	Adenocarcinoma 26 (35.6%)<br>Melanoma 16 (21.9%)<br>Esthesioneuroblastoma 13 (17.8%)<br>Adenoid cystic carcinoma 7 (9.6%)<br>SCC 6 (8.2%)<br>Undifferentiated carcinoma 5 (6.9%)	Nasal cavity 45 (61.6%)<br>Sphenoethmoidal area 15 (20.5%)<br>Olfactory cleft 5 (6.8%)<br>Maxillary sinus 3 (4.1%)<br>Other areas 5 (7.0%)
5	Eisen M.D. et al.[25]	Basal-cell carcinoma (1)<br>Adenocarcinoma (1)<br>Esthesioneuroblastoma (1)<br>Chondrosarcoma (1)	Frontal sinus (1)<br>Ethmoidal sinus (1)<br>Cribriform plate (1)<br>Nasal septum (1)
6	Geltzeiler M. et al.[26]	ONB	Nasal cavity
7	Ishida H. et al.[14]	Basaloid squamous-cell carcinoma (1)<br>SCC (4)	Ethmoidal sinus (3)<br>Maxillary sinus (1)<br>Nasal cavity (1)

Notes:
SCC—squamous-cell carcinoma
ACC—adenoid cystic carcinoma
BCC—basal-cell carcinoma
SNUC—sinonasal undifferentiated carcinoma
INI-1-deficient carcinoma—integrase-interactor-1-deficient carcinoma
NOS—not otherwise specified
ONB—olfactory neuroblastoma

Table S7. Summary of the sinonasal tumors' subsites with orbital and/or intracranial invasion.

First authors	Salfrant M. et al.[7]	Ferrari M. et al.[16]	Meerwein C.M. et al.[15]	Eisen M.D. et al.[25]	Geltzeiler M. et al.[26]	Ishida H. et al.[14]	Summary
**Number of patients with orbital and/or IC invasion**	176 (but MRI available for 160 pat-s)	**123**	**73**	**4**	**209**	**3**	**588**
**Tumor localization**							-
Ethmoid	155 (88%)			1 (25%)		3 (100%)	**159**
Olfactory cleft/cribriform plate	13 (7%)		5 (6.8%)	1 (25%)	209 (100%)		**228**
Nasal septum	3 (2%)			1 (25%)			**4**
Frontal	1 (0.6%)			1 (25%)			**2**
Maxillary	1 (0.6%)	65 (52.8%)	3 (4.1%)				**69**
Orbit	1 (0.6%)						**1**
Sphenoid	1 (0.6%)						**1**
Nasoethmoidal		58 (47.2%)					**58**
Nasal cavity	1 (0.6%, middle turbinate)		45 (61.6%)				**46**
Sphenoethmoidal area			15 (20.5%)				**15**
Other			5 (7.0%)				**5**

Notes:
IC—intracranial

Table S8. Technical details of the MRI vendors and MRI protocols in the studies.

First authors	Manufacturer and model	Field strength	Coil	Contrast injection, agent	MRI protocols	MRI sequences	Slice thickness (mm)	Gap (mm)	FOV (cm)	Matrix size
McIntyre J.B. et al.[6]	-	-	-	-	-	-	-	-	-	-
Salfrant M. et al.[7]	-	-	-	-	-	-	-	-	-	-

Ferrari M. et al.[16]	Siemens Avanto and Siemens Area, Erlangen, Germany	1,5 T	head coil	-	standard acquisition protocol	axial and coronal turbo spin echo (TSE) T2, in the most appropriate plane TSE T1, axial DWI, postGad fat-suppressed axial three-dimensional gradient echo T1 (VIBE)	3 mm for precontrast and 0,6 mm for postcontrast VIBE images	-	-	512 × 256
Meerwein C.M. et al.[15]	-	1,5 T or 3,0 T	-	-	minimal pulse sequence set	axial and coronal T2-WI with and without fat saturation, axial and coronal T1-WI (with and without contrast enhancement and fat saturation), DWI	-	-	-	-
Eisen M.D. et al.[25]	GE Medical Systems, Milwaukee, Wis	1,5 T	volume neck or quadrature head coil	0.1 mmol/kg	-	sagittal T1-WI, axial T1-WI, fast spin echo T2-WI, postGad axial T1-WI	-	-	-	256 × 192
Geltzeiler M. et al.[26]	-	-	-	-	-	-	-	-	-	-
Ishida H. et al.[14]	Gyroscan (Philips)	1,5 T	-	-	-	T1, T2, and Gadolinium (Gd)-enhanced T1-WI	5	-	-	-

Notes:
"-" signifies missing data
FS—fat-suppressed
Gad, Gd—Gadolinium
TSE—turbo spin echo
DWI—diffusion-weighted imaging
VIBE—volumetric interpolated breath-hold examination.

Table S9. MRI protocols used in the studies.

**Total n = 4**	**Sequences**	**Slice thickness, TR, TE**
**Ferrari M. et al.**[16]	axial and coronal turbo spin echo (TSE) T2	3 mm, -
	in the most appropriate plane TSE T1	3 mm, -
	axial DWI	3 mm, -
	postGad fat-suppressed axial three-dimensional gradient echo T1 (VIBE)	0,6 mm, -
**Meerwein C.M. et al.**[15]	axial and coronal T2-WI with and without fat saturation	-
	axial and coronal T1-WI (with and without contrast enhancement and fat saturation)	-
	DWI	-
**Eisen M.D. et al.**[25]	sagittal T1-WI	400–600 ms, 11–17 ms
	axial T1-WI	500–700 ms, 11–20 ms
	fast spin echo T2-WI	3000–5000 ms, 80–108 ms

	postGad axial T1-WI	500–700 ms, 11–20 ms
**Ishida H. et al.**[14]	axial, coronal, and sagittal scanning planes were selected	
	T1 WI	5 mm, 520–595 ms, 12–20 ms
	T2 WI	5 mm, 3,000–4,500 ms, 100 ms
	Gadolinium-enhanced T1-WI	5 mm, 520–595 ms, 12–20 ms
**Salfrant et al.**[7]	T2 in axial and coronal planes	-
	contrast-enhanced T1 with fat saturation in axial and coronal planes	-

Notes:
"-" signifies missing data
TE—echo time, TR—repetition time, TSE—turbo spin echo, DWI—diffusion-weighted imaging, VIBE—fat-suppressed axial three-dimensional gradient echo T1 (volumetric interpolated breath-hold examination).

Table S10. Summary of the histological forms of sinonasal tumors in the studies.

First authors	McIntyre J.B. et al.[6]	Salfrant M. et al.[7]	Ferrari M. et al.[16]	Meerwein C.M. et al.[15]	Eisen M.D. et al.[25]	Geltzeiler M. et al.[26]	Ishida H. et al.[14]	Summary
**Number of patients with orbital and/or IC invasion who had reference standard test**	**39**	176 (but MRI available for **160** pat-s)	**123**	**73**	**4**	**209**	**3**	**627**
**Tumor histology**								
SCC	9 (23%)	10 (6%)	45 (36.6%)	6 (8.2%)			2 (67%)	**72**
ACC	3 (7.7%)		14 (11.3%)	7 (9.6%)				**24**
Adenocarcinoma	9 (23%)		20 (16.3%)	26 (35.6%)	1 (25%)			**56**
Mucosal melanoma	4 (10%)	9 (5%)	10 (8%)	16 (21.9%)				**39**
ONB	4 (10%)	36 (20%)	5 (4%)	13 (17.8%)	1 (25%)	209 (100%)		**268**
Salivary duct carcinoma			1 (0.8%)					**1**
Epithelial-myoepithelial carcinoma			1 (0.8%)					**1**
Myoepithelial carcinoma			1 (0.8%)					**1**
Polymorphous adenocarcinoma			1 (0.8%)					**1**

Mesenchymal tumors			7 (5.7%)				**7**
SNUC	3 (7.7%)		4 (3.2%)	5 (6.9%)			**12**
Clear-cell carcinoma	1 (2.6%)						**1**
Chondrosarcoma	1 (2.6%)				1 (25%)		**2**
Chordoma	1 (2.6%)						**1**
Osteosarcoma	1 (2.6%)						1
Metastatic-renal-cell carcinoma	1 (2.6%)						**1**
Sarcoma	1 (2.6%)						**1**
Spindle-cell carcinoma	1 (2.6%)						**1**
Intestinal-type adenocarcinoma		72 (41%)					**72**
Non-intestinal-type adenocarcinoma		28 (16%)					**28**
Neuroendocrine carcinoma		6 (3%)					**6**
Rhabdomyosarcoma		3 (2%)					**3**
Carcinoma NOS			5 (4%)				**5**
BSS			3 (2.4%)				**3**
Basaloid squamous-cell carcinoma			2 (1.6%)		1 (25%)		**3**
INI-1-deficient carcinoma						1 (33%)	**1**
Teratocarcinosarcoma			2 (1.6%)				**2**
Other			2 (1.6%)				**2**
		12 (7%)					**12**

Notes:
SCC—squamous-cell carcinoma
ACC—adenoid cystic carcinoma
BCC—basal-cell carcinoma
SNUC—sinonasal undifferentiated carcinoma
INI-1-deficient carcinoma—integrase-interactor-1-deficient carcinoma
NOS—not otherwise specified
ONB—olfactory neuroblastoma
